# Corrugation at the Trailing Edge Enhances the Aerodynamic Performance of a Three-Dimensional Wing During Gliding Flight

**DOI:** 10.3390/biomimetics10050329

**Published:** 2025-05-17

**Authors:** Kaipeng Li, Na Xu, Licheng Zhong, Xiaolei Mou

**Affiliations:** 1 School of Electromechanical and Automotive Engineering, Yantai University, Yantai 264005, China; likaipeng@s.ytu.edu.cn (K.L.); zhonglicheng@s.ytu.edu.cn (L.Z.); 2 School of Civil Engineering, Yantai University, Yantai 264005, China; mouxiaolei@ytu.edu.cn

**Keywords:** corrugated wing, gliding flight, number of corrugations, linear corrugation variation, chordwise corrugation placement

## Abstract

Dragonflies exhibit remarkable flight capabilities, and their wings feature corrugated structures that are distinct from conventional airfoils. This study investigates the aerodynamic effects of three corrugation parameters on gliding performance at a Reynolds number of 1350 and angles of attack ranging from 0° to 20°: (1) chordwise corrugation position, (2) linear variation in corrugation amplitude toward the trailing edge, and (3) the number of trailing-edge corrugations. The results show that when corrugation structures are positioned closer to the trailing edge, they generate localized vortices in the mid-forward region of the upper surface, thereby enhancing aerodynamic performance. Further studies show that a linear increase in corrugation amplitude toward the trailing edge significantly delays the shedding of the leading-edge vortex (LEV), produces a more coherent LEV, and reduces the number of vortices within the corrugation grooves on the lower surface. Consequently, the lift coefficient is maximized with an enhancement of 28.99%. Additionally, reducing the number of trailing-edge corrugations makes the localized vortices on the upper surface approach the trailing edge and merge into larger, more continuous LEVs. The vortices on the lower surface grooves also decrease in number, and the lift coefficient is maximally increased by 20.09%.

## 1. Introduction

Owing to their maneuverability, micro air vehicles (MAVs) have diverse applications in environmental monitoring, disaster relief, and other civilian fields [1]. Currently, many MAV wings are designed to imitate those of large aircraft; however, their operating Reynolds numbers (*Re* = 10^3^~10^5^) are significantly lower than those of conventional aircraft. Thus, this discrepancy suggests that wings designed for large-scale vehicles may not be suitable for MAVs [2], Therefore, it is very necessary to develop a new type of wing specially designed for MAVs. Compared to other insects and birds, dragonflies are capable of continuous gliding with superior flight performance [3], making them an ideal reference for MAV wing design. A remarkable feature of dragonfly wings is their corrugated structure, which not only improves the stiffness of the wings [3,4,5] but also offers superior aerodynamic performance [6,7]. Consequently, investigating how corrugated structures influence the gliding performance of three-dimensional wings is critical for improving MAV efficiency.

The aerodynamic effects of corrugated structures were first studied by Rees [8] at a Reynolds number (*Re*) of 450~900. The investigation showed that airflow was trapped within the grooves of corrugations, forming vortices that stagnated or rotated slowly, making the corrugated wing more streamlined without significant aerodynamic losses compared to smooth airfoils. Research by Newman [9] reached a similar conclusion. However, Buckholz [10] experimentally demonstrated that corrugated structures could increase the lift of dragonfly wings at *Re* = 1500. Wakeling and Ellington [11] tested real dragonfly wings and further confirmed that a corrugated structure improves lift. Kesel [7] conducted experiments with three different spanwise sections of dragonfly wings at *Re* = 7880 and 10,000, finding that the aerodynamic performance of different corrugated airfoils varied, with negative pressure forming in the corrugation grooves, resulting in greater lift compared to flat plates. Subsequently, Levy and Seifert [12] conducted a comparative study on corrugated and smooth airfoils at *Re* = 2000 to 8000 and found that corrugated airfoils outperformed traditional streamlined airfoils. More recently, Narita [13] used 3D scanning technology to reconstruct real dragonfly hindwing models and found through numerical simulations that the corrugated structures of real wings exhibited better aerodynamic performance compared to flat plate wings with corrugations only near the leading edge. Giorgio [14] combined corrugation with a flexible surface and found that this combination effectively improved the aerodynamic performance of the model wing, increasing it by approximately 50%.

Dragonfly wings feature continuous corrugated structures along the chordwise direction [15], with varying effects on the flow field depending on their chordwise position [16]. However, the influence of chordwise corrugation position on aerodynamic performance remains less studied. Chandra [17] experimentally compared corrugated structures near the leading edge and trailing edge, finding that lift increased significantly when corrugated structures were positioned near the trailing edge, likely due to the reattachment of the LEV to the trailing edge. Xu [18] and Rohit [19] supported these findings through numerical simulations at a low *Re* (*Re* ≤ 2300). Other studies have focused on single-placement factors of a corrugated structure near the leading edge or trailing edge. These studies also showed that different corrugated chordwise positions have a greater effect on aerodynamic performance. For instance, Meng [20] investigated the effect of a corrugated structure close to the leading edge on the aerodynamic performance of the gliding at *Re* = 200~2400 and found that the leading-edge corrugated structure reduced lift due to stronger vortices within the lower-surface grooves, resulting in a localized low-pressure region on the lower surface and greater separation of the LEV from the upper surface, thereby reducing upper-surface suction. Mukherjee [21] incorporated corrugations near the trailing edge of triangular wings and found that the trailing-edge corrugated structure increased lift by a maximum of 27% at a high *Re* (*Re* = 10^5^). Guilarte Herrero [22] showed that airfoils with two leading-edge corrugations exhibited better aerodynamic performance at *Re* = 10,000 to 25,000. 

In nature, the amplitude of dragonfly corrugated structures decreases gradually from the front to the rear of the wing [23]. Zhang [24] and Shabbir [25] investigated the impact of corrugation amplitude on aerodynamic performance using two-dimensional airfoils. Zhang [24] varied the overall airfoil corrugation amplitude and found that reducing the corrugation amplitude improved lift and reduced drag, while Shabbir [25], based on the airfoil used by Murphy and Hu [26], altered only the amplitude of two leading-edge corrugations and reached similar conclusions to Zhang [24], finding that an increase in amplitude at most angles of attack (AOAs) caused strong vortices to appear in the leading-edge grooves, which reduced lift and increased drag, thus degrading the aerodynamic performance of the airfoil. Wang [27] extended this research to three-dimensional wings, indirectly adjusting corrugation amplitude by varying corrugation angles, and found that smaller angles (larger amplitudes) resulted in poorer aerodynamic performance when the corrugation angle was less than 120°.

The corrugated structure of dragonfly wings is mainly concentrated in the wing root area, and the number of corrugations gradually decreases along the wingspan [23]. However, the effect of the number of corrugations has been less studied. Rohit [19] investigated the effect of changing the number of leading-edge corrugations at *Re* = 1000 and found that decreasing the number of corrugations at the leading edge increased the lift-to-drag ratio of the airfoil.

Table 1 presents a comparison between this study and prior research on corrugated wings. It can be observed that most of the previous studies on the effect of corrugation chordwise position on aerodynamic performance focus on two-dimensional airfoils or only consider single-placement factors. Similarly, most studies on corrugation amplitude have mainly involved scaling the overall amplitude or altering a few corrugation amplitudes of two-dimensional airfoils. However, real dragonfly wing corrugations exhibit a gradual variation, and studies on the aerodynamic performance of gradually varying corrugations are rarely conducted. Fewer studies have been conducted on the effect of the number of corrugations on aerodynamic performance. Addressing these gaps, this paper investigates the effect of the chordwise position on the aerodynamic performance of three-dimensional corrugated wings at the *Re* corresponding to real dragonfly gliding. Additionally, this study explores the effects of linearly varying corrugation structures and the number of corrugations on aerodynamic performance, building on computational results related to corrugation chordwise position.

## 2. Materials and Methods

### 2.1. Wing Model Design

**Figure 1:**
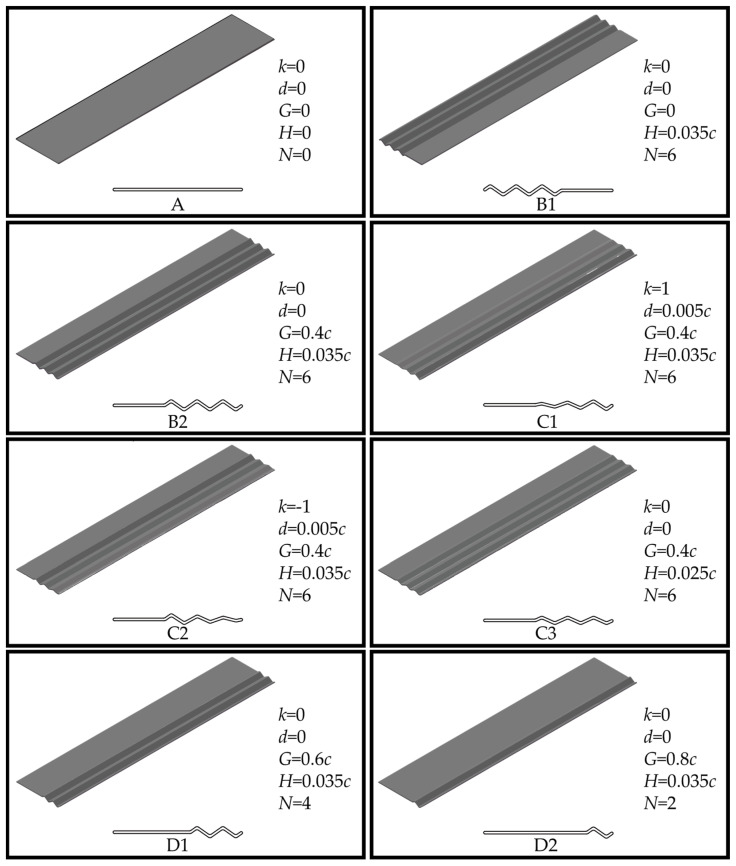
Geometric configurations and parameters of model wings with varying corrugation features.

In this paper, we study a flat plate wing and seven corrugated wings with different corrugation configurations. All model wings feature a rectangular planform and an aspect ratio *AR* = *R*/*c* = 5. Here, *R* refers to the wingspan, while *c* denotes the chord length (*c* = 10 mm in this study). This aspect ratio approximates the average value observed in dragonfly forewings [28]. To isolate the aerodynamic effects of the corrugation structure, all model wings maintain a uniform thickness of 0.02*c*. As presented in Figure 1, the flat plate wing is labeled as A, while the seven corrugated wings are labeled as B1, B2, C1, C2, C3, D1, and D2. 

**Figure 2:**
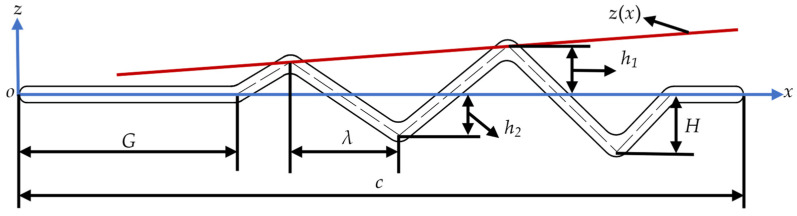
Schematic diagram of corrugated wing section parameters (o is the origin of the coordinates).

The corrugation structures for the corrugated wings are based on the triangular wave model proposed by Rees [29]. Specifically, the corrugated structures of B1 and B2 are positioned near the leading and trailing edges of the wing, respectively. The corrugation amplitudes of wings C1 and C2 increase and decrease toward the trailing edge, respectively, with both wings having the same mean amplitude value. Based on B2, the amplitude is further adjusted to create C3, whose amplitude value is equal to the average value of C1 or C2. Wings D1 and D2 are created by reducing 2 and 4 triangular wave structures, respectively, based on B2. As illustrated in Figure 2, to parameterize the corrugated structure more conveniently, the midline of the cross-sectional profile of the corrugated wings is given by the equation for *z*(*x*):

(1) $$z(x)=k\frac{d}{\lambda }(x+\frac{k(G+\lambda -c)+c-2N\lambda -3G}{2}+\frac{kH\lambda }{d+0^{d}})+0^{d}H$$

where *G* represents the distance of the corrugation structure from the leading edge; *λ* denotes the wavelength of the triangular wave; *H* is the maximum amplitude of the triangle wave in the corrugation structure; *d* represents the absolute value of the amplitude difference between adjacent triangular waves, defined as $d=\left|h_{1}-h_{2}\right|$. The parameter *k* is the corrugation amplitude variation coefficient, depending on the rule of change in the corrugation amplitude of the wing. It is set to 1 when the corrugation amplitude increases linearly toward the trailing edge, −1 when it decreases linearly toward the trailing edge, and 0 when there is no change in the corrugation amplitude. *N* represents the number of triangular waves that make up the corrugated structure. Once these parameters are determined, the midline of the corrugated wing can be obtained by sequentially connecting the coordinate points in Equation (2).

(2) $$\left(0,0),(G,0),\underset{n}{\underset{︸}{(G+(0.5+n-1)\lambda ,{\left(-1\right)}^{n-1}z),\cdots }},(G+N\lambda ,0),(c,0\right)$$

Here, *n* is an integer from 1 to *N*, with each value corresponding to a coordinate point. In SolidWorks 2022, the midline is extended equidistantly on both sides to form corrugated airfoils. The corners, leading edge, and trailing edge of the corrugated airfoils are smoothly transitioned with arcs. The corrugated airfoils are then stretched along the spanwise direction to form the three-dimensional corrugated wings used in this study. Similar to previous studies using corrugated wing structures [30,31,32,33,34], all corrugated wavelengths λ=0.1c and other parameters are labeled in Figure 1.

### 2.2. The Flow Equations and Solution Method

In this study, Fluent Meshing was used for mesh generation, and Fluent 24.0 was employed for numerical simulations of the model wings. Given that dragonflies operate at a low *Re*, the simulations utilized a laminar flow model to solve the unsteady, incompressible Navier–Stokes (N-S) equations [13,35,36,37]. The three-dimensional, dimensionless N-S equations are expressed as follows:

(3) $$\frac{\partial u}{\partial x}+\frac{\partial v}{\partial y}+\frac{\partial w}{\partial z}=0$$

(4) ∂u∂τ+u∂u∂x+v∂u∂y+w∂u∂z=−∂P∂x+1Re(∂2u∂x2+∂2u∂y2+∂2u∂z2)

(5) ∂v∂τ+u∂v∂x+v∂v∂y+w∂v∂z=−∂P∂y+1Re(∂2v∂x2+∂2v∂y2+∂2v∂z2)

(6) ∂w∂τ+u∂w∂x+v∂w∂y+w∂w∂z=−∂P∂z+1Re(∂2w∂x2+∂2w∂y2+∂2w∂z2)

Here, *u*, *v*, and *w* are the velocity components in the *x*, *y*, and *z* directions, respectively; *P* represents the fluid pressure; *τ* is the time; *Re* is the Reynolds number. A pressure-based solver in unsteady mode was used. The SIMPLE algorithm [18,37] was used to solve the velocity–pressure coupling equations. Second-order upwind schemes were applied for the discretization of convective fluxes in the momentum equations. The pressure terms were discretized using a second-order scheme, the transient terms were solved using a second-order implicit scheme, and the viscous terms were discretized using the default second-order central differencing scheme.

**Figure 3:**
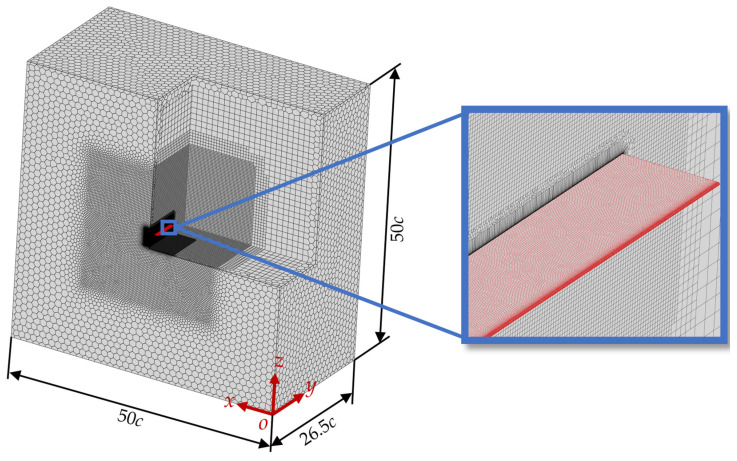
Computational domain and mesh division.

The computational domain and meshing are illustrated in Figure 3. The size of the computational domain is 50*c* × 26.5*c* × 50*c*, which ensures that the model wing is sufficiently far from the boundaries compared to other studies [13,38,39,40]. The boundary at *y* = 0 is a symmetric boundary condition, that is, $\frac{\partial P}{\partial y}=0$, $\frac{\partial u}{\partial y}=0$, $\frac{\partial w}{\partial y}=0$, *v* = 0; while *x* = 0 is a pressure outlet boundary condition, that is, *P* = *P*_0_, $\frac{\partial u}{\partial x}=0$, $\frac{\partial v}{\partial x}=0$, $\frac{\partial w}{\partial x}=0$, where $P_{0}$ denotes the standard atmospheric pressure. To minimize the influence of boundary conditions on the flow field, the remaining boundaries are set as velocity inlets along the negative *x*-axis direction [37,41], that is, $u=U_{\infty }$, v=0, w=0, *P* = *P*_0_, where $U_{\infty }$ is the freestream velocity. The surface of the model wing is set as a no-slip and impermeable wall, that is, u=0, v=0, w=0. The model wing is in the central region of the computational domain, with the wing root positioned 1.5*c* from the symmetry boundary. The Poly-Hexcore body mesh generation method was employed for meshing, enabling the co-nodal connection of hexahedral and polyhedral meshes to improve mesh quality. Additionally, the Body of Influence (BOI) method was used to refine the mesh around the model wing, ensuring a sufficiently fine mesh to capture flow variations while significantly reducing the number of cells and saving computational resources.

In this study, the air density is set to ρ = 1.225 kg/m^3^, and the Reynolds number (*Re*) is calculated using Equation (7):

(7) Re=cU∞v

where v is the kinematic viscosity of the fluid (v = 1.648 × 10^−5^ m^2^/s). The *Re* for dragonfly gliding flight typically ranges from 700 to 2400 [11], and in this study, the model wing glides at *Re* = 1350, with a freestream velocity of $U_{\infty }$ = 2.225 m/s. The aerodynamic lift coefficient $C_{L}$ and drag coefficient $C_{D}$ of the model wing are defined as

(8) $$C_{L}=\frac{F_{L}}{0.5\rho U_{\infty }^{2}S}$$

(9) $$C_{D}=\frac{F_{D}}{0.5\rho U_{\infty }^{2}S}$$

where $F_{L}$ is the model wing aerodynamic lift, $F_{D}$ is the aerodynamic drag, and *S* is the flat plate wing area (*S* = *Rc*), which is applied to all corrugated wings. The time-averaged lift coefficient ${\bar{C}}_{L}$ and time-averaged drag coefficient ${\bar{C}}_{D}$ are defined as

(10) $${\bar{C}}_{L}=\frac{\frac{1}{\tau }\int_{0}^{\tau }F_{L}d\tau }{0.5\rho U_{\infty }^{2}S}$$

(11) $${\bar{C}}_{D}=\frac{\frac{1}{\tau }\int_{0}^{\tau }F_{D}d\tau }{0.5\rho U_{\infty }^{2}S}$$

**Figure 4:**
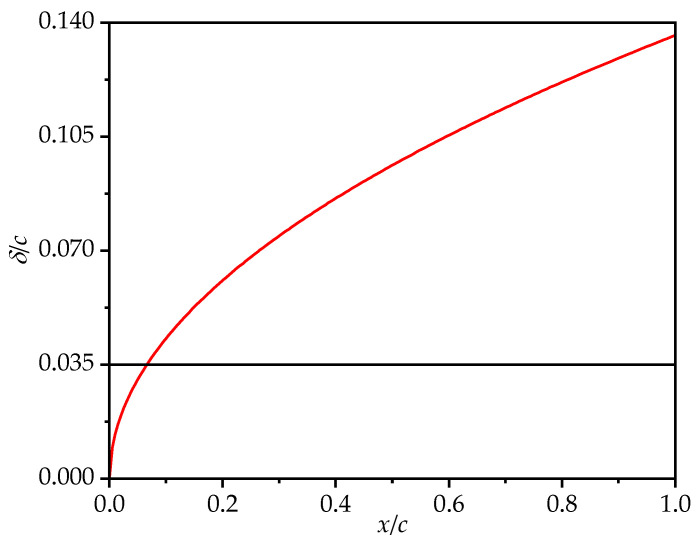
Comparison between the theoretical boundary layer thickness *δ* of the flat plate (red line) and the maximum corrugation amplitude (black line).

Since the Reynolds number in this study is *Re* = 1350 and a laminar flow model is employed, Figure 4 presents a comparison between the theoretical boundary layer thickness of the flat plate wing and the maximum corrugation amplitude (0.035*c*). The boundary layer thickness is calculated based on the Blasius solution. It can be observed that across most of the chordwise direction, the boundary layer thickness exceeds the maximum corrugation amplitude. Only in the region near the leading edge (approximately 0~0.1*c*) is the boundary layer thinner than the maximum corrugation amplitude, indicating that only the first peak of the leading-edge corrugation (B1) extends beyond the boundary layer. This further supports the appropriateness of using a laminar model for the simulation of the corrugated wing.

### 2.3. Verification and Validation

To begin with, a time step independence verification is conducted. In this study, since the gliding angle of attack (AOA) ranges from 0° to 20°, a relatively large AOA of 15° is selected for the verification. Table 2 presents the time-averaged lift coefficient (${\bar{C}}_{L}$), drag coefficient (${\bar{C}}_{D}$), and relative error ($\Delta {\bar{C}}_{L}$, $\Delta {\bar{C}}_{D}$) under different time steps. It can be observed that the differences in ${\bar{C}}_{L}$ and ${\bar{C}}_{D}$ among the three time steps are minimal and further diminish as the time step decreases. When the time step is 1 × 10^−4^ s, compared with the time step of 5 × 10^−5^ s, the ${\bar{C}}_{D}$ values are exactly the same, and the ${\bar{C}}_{L}$ values differ by only 0.13%. Considering computational efficiency, a time step of 1 × 10^−4^ s is adopted in this study, consistent with the time step used by Kim [36] and Chen [37] in their studies at a similar *Re* (*Re* = 1400).

**Figure 5:**
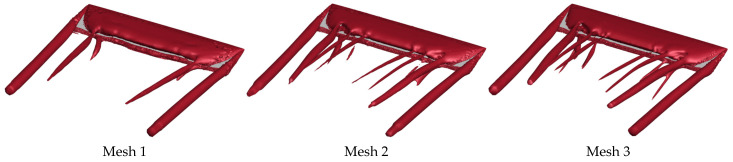
*Q*-criterion iso-surfaces for Mesh 1, Mesh 2, and Mesh 3 (α = 15°, *Re* = 1350, *Q* = 50,000).

Furthermore, a mesh independence verification was conducted to minimize the influence of the mesh quantity on the simulation accuracy. Based on the fluid domain of a flat plate wing, three sets of computational domains with different mesh sizes were constructed by varying the mesh cell size in the BOI refinement zone and the thickness of the first-layer mesh. Mesh 1 has 2.1 million cells, with a first-layer mesh thickness of 0.002*c*; Mesh 2 has 5 million cells, with a first-layer mesh thickness of 0.001*c*; and Mesh 3 has 10 million cells, with a first-layer mesh thickness of 0.001*c*. The time-averaged lift coefficient (${\bar{C}}_{L}$) and drag coefficient (${\bar{C}}_{D}$) calculated for the three sets of meshes were evaluated, as shown in Table 3. For the flat plate wing at an AOA of α = 15°, the relative error of the ${\bar{C}}_{L}$ gradually decreased with increasing mesh density, with the maximum error declining from 4.06% to 2.55%. Notably, results from Mesh 2 and Mesh 3 exhibited negligible differences. Flow field structures, visualized via Q-criterion iso-surfaces after time-averaging, are shown in Figure 5. From the figure, it can be observed that Mesh 1 results deviate significantly from Meshes 2 and 3, whereas Mesh 2 shows only a minor difference compared to Mesh 3. To balance computational accuracy and efficiency, Mesh 2 was chosen for simulations in this study.

**Figure 6:**
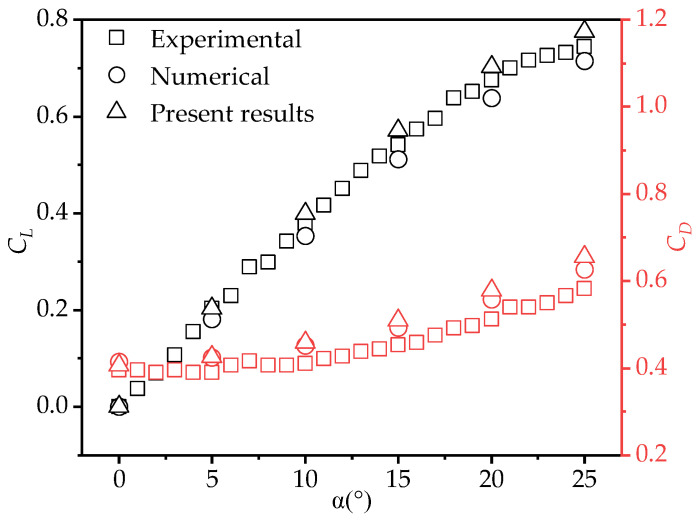
Comparison of simulation results in this study with Taira’s experimental results [42] and Meng’s simulation results [20] (*AR* = 2, *Re* = 100).

Lastly, the reliability of the numerical method was verified by comparing the simulation results with experimental data [42] and prior computational studies [20]. Using the same model as in Taira’s experiment [42], the lift coefficients ($C_{L}$) and drag coefficients ($C_{D}$) of the flat plate wing (*AR* = 2) were simulated under conditions of *Re* = 100 at various AOAs. As shown in Figure 6, the simulation results in this study exhibit a consistent trend with Taira’s experimental results [42] and Meng’s simulation results [20]. The calculated force coefficients at different AOAs also show good agreement. This indicates that the numerical method and mesh scale used are reliable and capable of accurately simulating the aerodynamic characteristics of gliding wings.

## 3. Results and Discussion

In this section, the aerodynamic performance of the three-dimensional wings during gliding is investigated in detail for *Re* = 1350 and an AOA (α) ranging from 0° to 20° (in increments of 5°). Based on the simulation results of eight model wings with different corrugation features, the effects of chordwise corrugation position, linear corrugation amplitude variation toward the trailing edge, and the number of trailing-edge corrugations on aerodynamic performance are analyzed.

### 3.1. Effect of Chordwise Corrugation Position

To examine the influence of chordwise corrugation position on the gliding performance of three-dimensional wings, the aerodynamic characteristics of the flat plate wing A are compared with those of corrugated wings B1 and B2. As illustrated in Figure 1, the corrugation structure of B1 is positioned near the leading edge, while that of B2 is located near the trailing edge.

**Figure 7:**
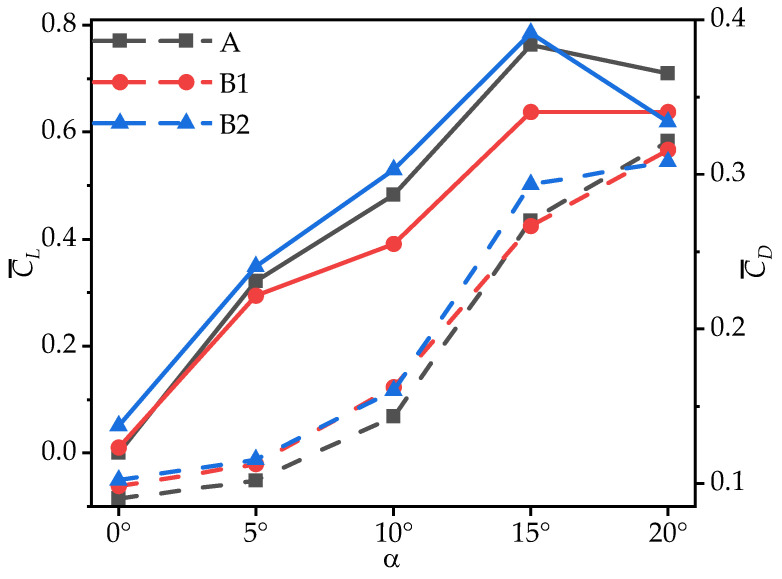
Variation in time-averaged lift coefficients (${\bar{C}}_{L}$, solid lines) and drag coefficients (${\bar{C}}_{D}$, dashed lines) of wings A, B1, and B2 with the angle of attack (AOA).

Figure 7 presents the time-averaged lift coefficient (${\bar{C}}_{L}$) and drag coefficient (${\bar{C}}_{D}$) of model wings A, B1, and B2 at various AOAs. It can be observed that, as the AOA increases, the ${\bar{C}}_{L}$ values of model wings A and B2 initially increase and then decrease, and ${\bar{C}}_{L}$ for corrugated wing B1 consistently increases (with minimal difference at α = 15° and 20°). The ${\bar{C}}_{L}$ values for B1 are significantly lower than those of wing A at most AOAs, exceeding wing A only marginally at α = 0°. In contrast, B2 exhibits higher ${\bar{C}}_{L}$ values than wing A at most AOAs, with a sharp decrease observed only at α = 20°, where it becomes the lowest among the three wings. Table 4 shows the corresponding rates of change in ${\bar{C}}_{L}$, ${\bar{C}}_{D}$, and the lift-to-drag ratio (${\bar{C}}_{L}/{\bar{C}}_{D}$) for corrugated wings B1 and B2 with respect to the flat plate wing A. At α = 10°, the ${\bar{C}}_{L}$ of B1 is 19.11% lower than that of wing A, while B2 is 9.70% higher than A, showing the largest difference in ${\bar{C}}_{L}$ between the corrugated wings and the flat plate wing. At α = 15°, the ${\bar{C}}_{L}$ of B2 reaches its maximum value of 0.785, slightly higher than that of wing A, while B1 remains 16.48% lower than wing A. These results indicate that, at most AOAs, corrugations near the leading edge (B1) significantly reduce the time-averaged lift coefficient compared to flat plate wing A, whereas corrugations near the trailing edge (B2) enhance lift generation. These results are consistent with the two-dimensional simulation findings of Xu [18] and the experimental results of Chandra [17].

The time-averaged drag coefficients (${\bar{C}}_{D}$) of model wings A, B1, and B2, along with the rates of change in ${\bar{C}}_{D}$ for wings B1 and B2 relative to wing A, are also analyzed. The observations show that the ${\bar{C}}_{D}$ values for all three wings increase with an increase in the AOA. At most AOAs, the ${\bar{C}}_{D}$ of B2 is significantly higher than that of wing A, with increases ranging from 8.74% to 13.61%. The only exception occurs at α = 20°, where the ${\bar{C}}_{D}$ of B2 is 4.09% lower than that of wing A. At small AOAs (α ≤ 10°), the ${\bar{C}}_{D}$ values of corrugated wings B1 and B2 are nearly identical but significantly higher than those of wing A, with the largest increase of 13.00% at α = 10°. At large AOAs (α ≥ 15°), the ${\bar{C}}_{D}$ of B1 is slightly lower than that of wing A, with differences within 2%.

**Figure 8:**
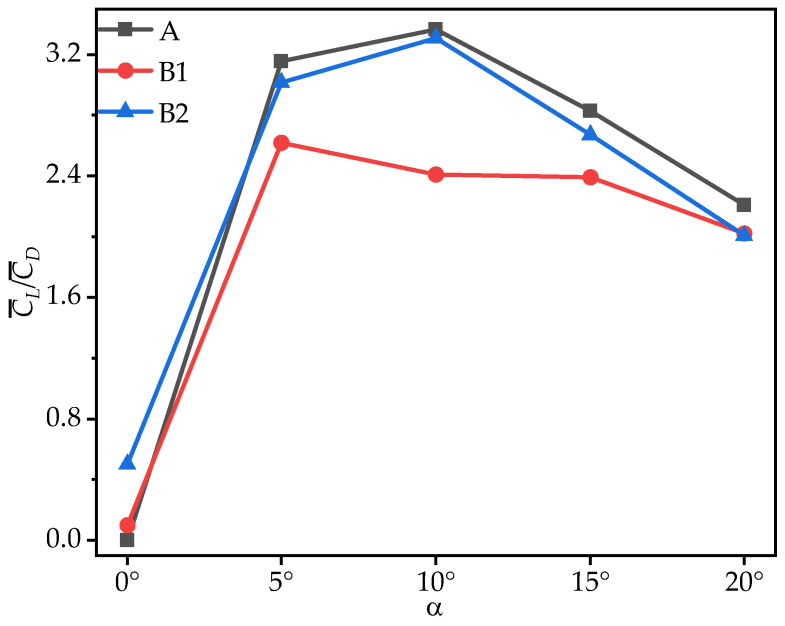
Variation in time-averaged lift-to-drag ratios (${\bar{C}}_{L}/{\bar{C}}_{D}$) of wings A, B1, and B2 with the angle of attack (AOA).

To comprehensively evaluate the aerodynamic performance of the model wings during gliding, Figure 8 presents the variation in the time-averaged lift-to-drag ratio (${\bar{C}}_{L}/{\bar{C}}_{D}$) with the AOA for wings A, B1, and B2. As shown in Figure 8 and Table 4, all three wings exhibit a consistent trend in ${\bar{C}}_{L}/{\bar{C}}_{D}$, which initially increases and then decreases as the AOA increases. At most AOAs, the ${\bar{C}}_{L}/{\bar{C}}_{D}$ of B1 is significantly lower than that of wing A, with a maximum reduction of 28.41%. For B2, the ${\bar{C}}_{L}/{\bar{C}}_{D}$ is slightly lower than that of wing A, with the difference ranging from 1.70% to 9.13%. Notably, the ${\bar{C}}_{L}/{\bar{C}}_{D}$ values of B1 and B2 exceed that of wing A only at α = 0°. This is similar to the two-dimensional results of Xu [18], where corrugation structures located at the trailing edge exhibit higher ${\bar{C}}_{L}/{\bar{C}}_{D}$ values than those located at the leading edge.

**Figure 9:**
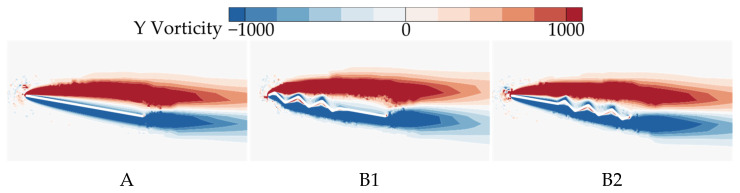
Time-averaged vorticity contours of wings A, B1, and B2 at 50% wingspan (α = 10°).

**Figure 10:**
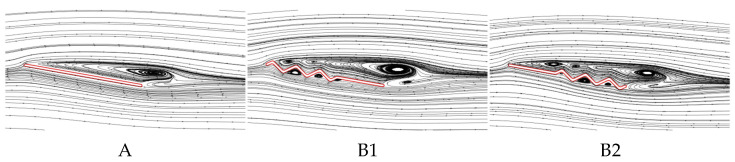
Time-averaged streamlines of wings A, B1, and B2 at 50% wingspan (α = 10°).

From the above analysis, it follows that B1 exhibits the poorest aerodynamic performance at most AOAs, with the lowest ${\bar{C}}_{L}$. In contrast, B2 shows aerodynamic performance closest to that of wing A and achieves the highest ${\bar{C}}_{L}$. To investigate the underlying reasons for the differences in gliding performance caused by varying chordwise corrugation positions, the time-averaged vorticity contours and streamline diagrams at 50% wingspan for wings A, B1, and B2 are compared at α = 10°, where the aerodynamic force difference is most pronounced, as shown in Figure 9 and Figure 10.

For the time-averaged vorticity contours in Figure 9, it is observed that flow separation occurs for all three wings, with the LEV showing signs of shedding. Among them, B1 generates the largest LEV. However, compared to A and B2, the LEV of B1 separates farther from the upper wing surface, limiting its lift generation. Although B2 exhibits the highest degree of LEV detachment, its LEV size is comparable to that of B1, and the LEV adheres more closely to the upper surface in the front-to-mid region (0~0.5*c*). The streamlines in Figure 10 reveal that on the lower surface of B1 and B2, airflow passing through the first corrugation generates a vortex in the subsequent corrugation groove. On the upper surface, flow separation occurs at the leading edge for A and B2, while for B1, separation begins at the first corrugation peak (located at 0.1*c* from the leading edge). Additionally, B2 generates a local vortex between the leading edge and the first corrugation, which enhances the negative pressure on the upper surface.

**Figure 11:**
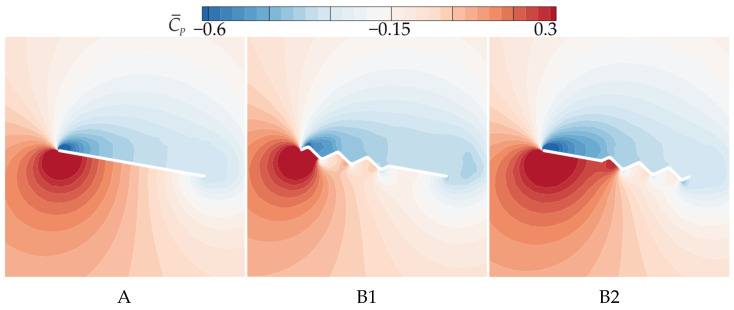
Time-averaged pressure coefficient contours of wings A, B1, and B2 at 50% wingspan (α = 10°).

**Figure 12:**
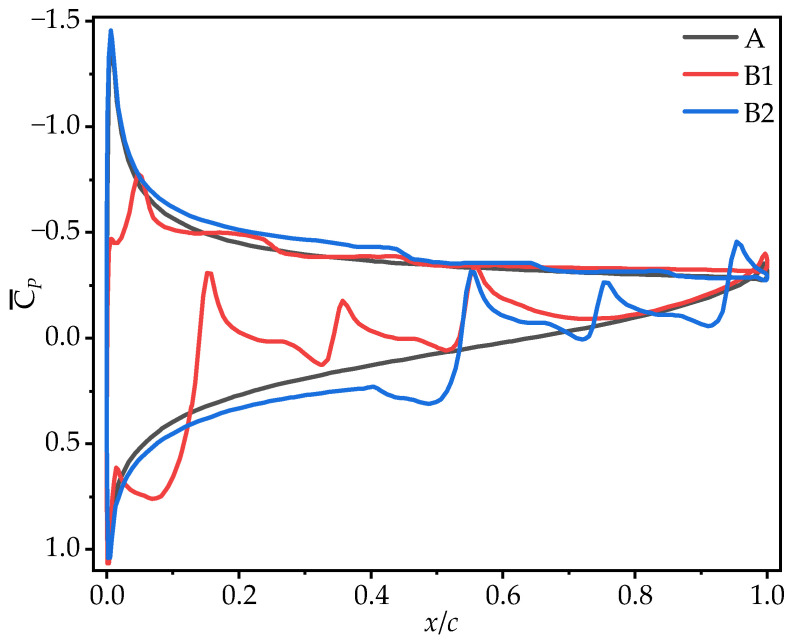
Time-averaged pressure coefficient curves of wings A, B1, and B2 at 50% wingspan (α = 10°).

To further clarify the effect of chordwise corrugation position on gliding performance, Figure 11 shows the time-averaged pressure coefficient (${\bar{C}}_{P}$) contour plots, and Figure 12 presents the corresponding ${\bar{C}}_{P}$ curves. On the upper surface, the local vortex between the leading edge and the first corrugation in B2 generates a larger negative pressure in the front-to-mid region (0~0.5*c*). In contrast, B1 exhibits significantly lower negative pressure than that of A and B2 in the 0~0.2*c* region due to the LEV separation at 0.1*c* from the leading edge, which shifts the center of the negative pressure on the upper surface. On the lower surface, the corrugation structures at different chordwise positions produce similar effects on pressure distribution, creating a positive pressure peak in the first corrugation groove (B1: 0~0.1*c*, B2: 0.4~0.5*c*) and negative pressure peaks at the subsequent corrugations. The corrugation structure near the leading edge (B1) produces the smallest positive pressure region on the lower surface, with a sharp pressure drop at the corrugations. This is likely the primary reason for B1’s poorest aerodynamic performance. In contrast, the corrugation structure near the trailing edge (B2) generates the largest positive pressure region on the lower surface, with a smaller pressure drop at the corrugations. This results in a greater pressure difference between the upper and lower surfaces for B2, explaining its highest time-averaged lift coefficient.

### 3.2. Effect of Linear Variation in Corrugation Amplitude

Based on the aerodynamic performance analysis of wings A, B1, and B2, the trailing-edge corrugation wing (B2) exhibits the highest ${\bar{C}}_{L}$, and gliding performance comparable to that of the flat plate wing (A). To further investigate the influence of chordwise variation in corrugation structures, this section analyzes and compares the aerodynamic characteristics of wings B2, C1, C2, and C3. As shown in Figure 1, the corrugation amplitude of C1 increases linearly toward the trailing edge, while that of C2 decreases linearly in the same direction. C3 features a uniform corrugation amplitude equal to the average of C1 and C2.

**Figure 13:**
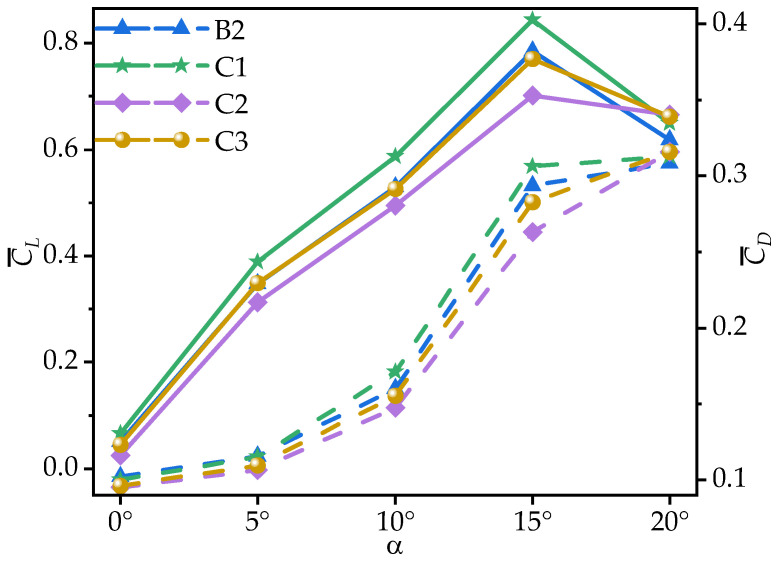
Variation in time-averaged lift coefficients (${\bar{C}}_{L}$, solid lines) and drag coefficients (${\bar{C}}_{D}$, dashed lines) of corrugated wings B2, C1, C2, and C3 with the angle of attack (AOA).

Figure 13 shows the time-averaged coefficients ${\bar{C}}_{L}$ and ${\bar{C}}_{D}$ of wings B2, C1, C2, and C3 at various AOAs. Table 5 provides the corresponding rates of change in the ${\bar{C}}_{L}$, ${\bar{C}}_{D}$, and time-averaged lift-to-drag ratios (${\bar{C}}_{L}/{\bar{C}}_{D}$) of wings C1, C2, and C3 relative to B2. From Figure 13 and Table 5, it can be seen that all wings exhibit an initial increase, followed by a decrease in ${\bar{C}}_{L}$, peaking at α = 15°. Within the range of α = 0°~15°, C1 achieves the highest ${\bar{C}}_{L}$, surpassing B2 by 5.06~28.99%, though the rate of change relative to B2 gradually decreases as the AOA increases. C2 exhibits the lowest ${\bar{C}}_{L}$, while the values of ${\bar{C}}_{L}$ for C3 are almost identical to those of B2. However, at α = 20°, C1, C2, and C3 exhibit similar ${\bar{C}}_{L}$ values, with C1, C2, and C3 showing a smaller stall, leading to a maximum improvement of 7.63% over B2.

From the ${\bar{C}}_{D}$ of the corrugated wings and the rates of change relative to B2, it can be observed that all corrugated wings exhibit the same trend in ${\bar{C}}_{D}$. Specifically, all wings show an increase in ${\bar{C}}_{D}$ with the AOA. The ${\bar{C}}_{D}$ values of C1 are generally similar to those of B2, with a slight increase (less than 5% at most AOAs), but 6.77% higher than those of B2 only at α = 10°. At most AOAs, the ${\bar{C}}_{D}$ of C2 is significantly lower than that of B2, with the greatest reduction of 10.39% at α = 15°, while the ${\bar{C}}_{D}$ of C3 is slightly lower than that of B2, with a maximum change of 5.87% at α = 0°.

**Figure 14:**
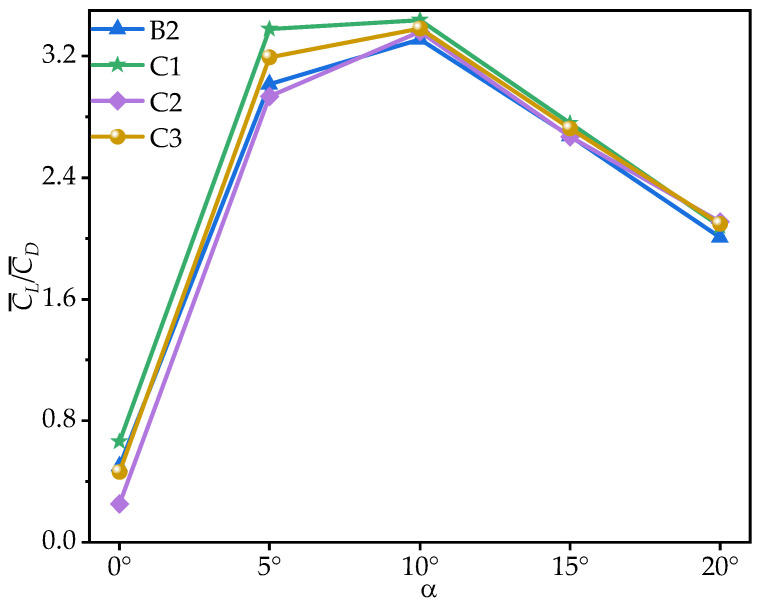
Variation in time-averaged lift-to-drag ratios (${\bar{C}}_{L}/{\bar{C}}_{D}$) of corrugated wings B2, C1, C2, and C3 with the angle of attack (AOA).

To further explore the effect of chordwise variation in trailing-edge corrugation on aerodynamic performance, Figure 14 presents the time-averaged lift-to-drag ratios (${\bar{C}}_{L}/{\bar{C}}_{D}$) of corrugated wings B2, C1, C2, and C3 at various AOAs. As shown in Figure 14 and Table 5, all corrugated wings follow a rise-then-decline trend in ${\bar{C}}_{L}/{\bar{C}}_{D}$, peaking at α = 10°. C1 achieves the highest ${\bar{C}}_{L}/{\bar{C}}_{D}$ across all AOAs. At most angles, the ${\bar{C}}_{L}/{\bar{C}}_{D}$ values of C2 and C3 are nearly identical to those of B2. The greatest difference between the corrugated wings C1, C2, C3, and B2 occurs at α = 0°, where C1 improves the gliding ratio by 31.96% compared to B2, while C2 and C3 reduce it by 49.77% and 8.03%, respectively.

**Figure 15:**
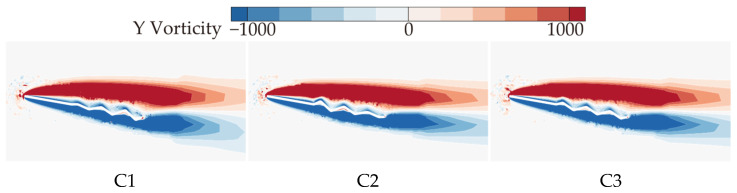
Time-averaged vorticity contours of corrugated wings C1, C2, and C3 at 50% wingspan (α = 10°).

**Figure 16:**
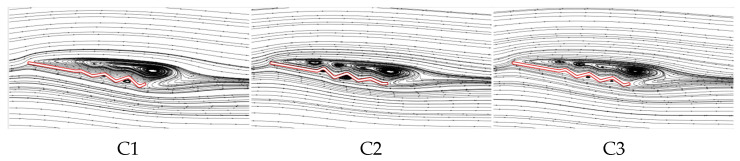
Time-averaged streamlines of corrugated wings C1, C2, and C3 at 50% wingspan (α = 10°).

It can be concluded that C1 exhibits the best aerodynamic performance and the highest ${\bar{C}}_{L}$. In contrast, C2 shows the lowest ${\bar{C}}_{L}$ at most AOAs, resulting in the poorest aerodynamic performance. This is similar to the conclusion in Xu’s study [18], where the corrugated structure is located in the middle of the airfoil. Comparing corrugated wings B2 and C3, it is evident that the ${\bar{C}}_{L}$, ${\bar{C}}_{D}$, and ${\bar{C}}_{L}/{\bar{C}}_{D}$ of B2 and C3 are similar at most AOAs. This suggests that the aerodynamic force differences of C1 and C2 are primarily due to the linear variation in the corrugation structures. To investigate the underlying reasons for the aerodynamic differences caused by chordwise variations in corrugation structures, the time-averaged vorticity contours and streamline diagrams at 50% wingspan for wings C1, C2, and C3 are compared at α = 10°, where the aerodynamic force difference is most pronounced, as shown in Figure 15 and Figure 16.

From the time-averaged vorticity contours shown in Figure 15 and Figure 9, it can be found that the extent of leading-edge vortex (LEV) shedding in C1, C2, and C3 is significantly lower than that of B2, with LEV sizes nearly identical across all wings. This suggests that the linear variation in the corrugation structure has a minimal effect on the size of the LEV. The streamlines in Figure 16 and Figure 10 reveal that, on the lower surface, the variation in chordwise amplitude results in significantly smaller vortices within the corrugation grooves for C1, C2, and C3 compared to B2, leading to a decrease in the negative pressure peak at the corrugations. On the upper surface, the localized vortex between the leading edge and the first corrugation disappears in C1 due to the smaller corrugation amplitude in the middle of C1. At the same time, larger and more continuous LEVs form in the mid-to-rear region (0.4*c*~*c*), which is advantageous for enhancing the negative pressure near the trailing edge. In contrast, for C2 and C3, similar to B2, local vortices are formed in the front-to-middle region, and their overall flow field structures are comparable to that of B2.

**Figure 17:**
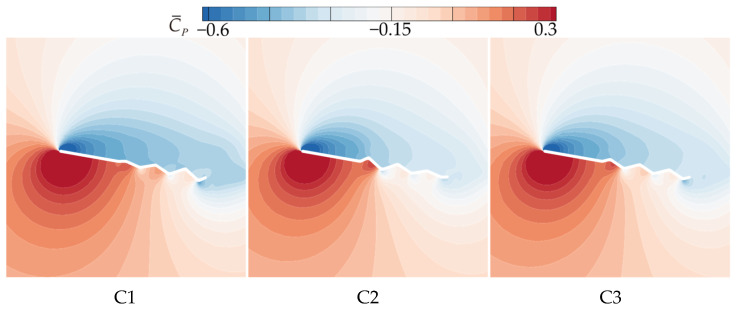
Time-averaged pressure coefficient contours of corrugated wings C1, C2, and C3 at 50% wingspan (α = 10°).

**Figure 18:**
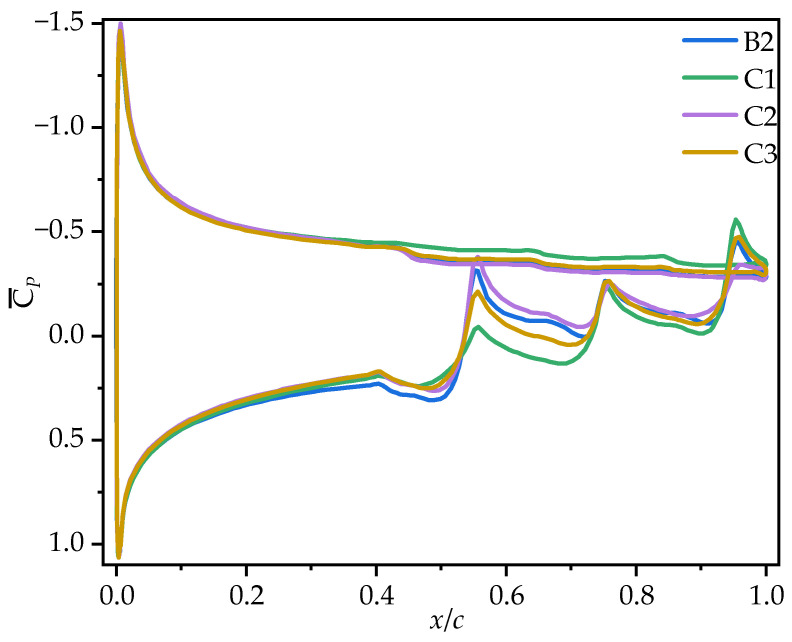
Time-averaged pressure coefficient curves of corrugated wings C1, C2, and C3 at 50% wingspan (α = 10°).

From the time-averaged pressure coefficient (${\bar{C}}_{P}$) contour plots in Figure 17 and the pressure coefficient curves in Figure 18, it can be observed that on the upper surface, the larger and more continuous LEV of C1 generates higher negative pressure in the mid-to-rear region (0.4*c*~*c*). The upper surface flow field structures of C2 and C3 are comparable to that of B2, resulting in comparable pressure coefficients on the upper surface. On the lower surface, the chordwise amplitude variation produces similar effects on pressure distribution, creating a positive pressure peak in the first corrugation groove (0.4*c*~0.5*c*) and negative pressure peaks at subsequent corrugations. The larger the amplitude in the middle region, the greater the positive pressure peak. C1, with the smallest corrugation amplitude in the middle region, generates significantly smaller vortices within the corrugation grooves compared to B2, which reduces the negative pressure peak at the corrugations. This results in an increased pressure difference between the upper and lower surfaces, leading to the optimal aerodynamic performance of C1. In contrast, C2, with a larger amplitude in the middle of the wing, produces the highest negative pressure peaks at the corrugations, resulting in the poorest aerodynamic performance. C3, with a uniform smaller amplitude than B2, weakens the vortices within the grooves, and the negative pressure peak is slightly smaller than that of B2, which explains why C3 exhibits slightly better aerodynamic performance than B2.

### 3.3. Effect of the Number of Trailing-Edge Corrugations

Based on B2, the previous section investigates the impact of chordwise variation in corrugation amplitude on aerodynamic performance. It is found that the wing with corrugation amplitude linearly increasing toward the trailing edge (C1) generates larger and more continuous vortices, which enhance negative pressure on the upper surface. On the lower surface, the strength and number of the vortices in the grooves are reduced, resulting in lower negative pressure peaks at the corrugations, making C1 have the optimal aerodynamic performance. This section further explores the effect of the number of corrugations on aerodynamic performance by comparing the corrugated wings B2, D1, and D2. As shown in Figure 1, D1 has two corrugations (four triangular waves), and D2 has one corrugation (two triangular waves).

**Figure 19:**
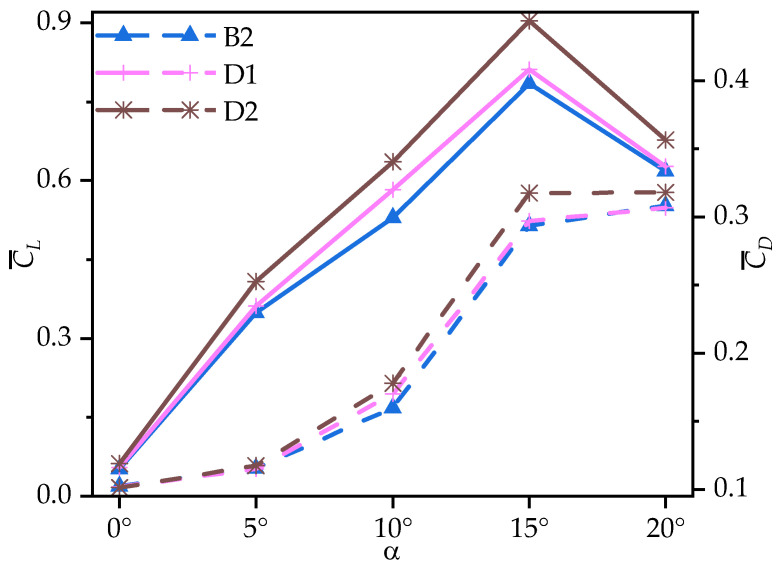
Variation in time-averaged lift coefficients (${\bar{C}}_{L}$, solid lines) and drag coefficients (${\bar{C}}_{D}$, dashed lines) of corrugated wings B2, D1, and D2 with the angle of attack (AOA).

Figure 19 presents the time-averaged lift coefficient (${\bar{C}}_{L}$) and drag coefficient (${\bar{C}}_{D}$) of corrugated wings B2, D1, and D2 at various AOAs. Table 6 provides the corresponding rates of change in the ${\bar{C}}_{L}$, ${\bar{C}}_{D}$, and time-averaged lift-to-drag ratios (${\bar{C}}_{L}/{\bar{C}}_{D}$) of wings D1 and D2 relative to B2. From Figure 19 and Table 6, it can be seen that all wings exhibit an initial increase, followed by a decrease in ${\bar{C}}_{L}$, peaking at α = 15°. Across all AOAs, the ${\bar{C}}_{L}$ increases as the number of corrugations decreases. Specifically, D1 shows a maximum enhancement of 10.00% in ${\bar{C}}_{L}$ compared to B2 at α ≤ 10°, with a slight increase at larger AOAs (α ≥ 15°). In contrast, D2 exhibits a significant increase in ${\bar{C}}_{L}$ compared to B2, with a maximum improvement of 20.09% at α = 10°.

Compared to the variation in the ${\bar{C}}_{L}$, the ${\bar{C}}_{D}$ changes of D1 and D2 are relatively smaller. While the ${\bar{C}}_{D}$ values of D1 and B2 are similar across all AOAs, D1 exhibits a maximum increase of 6.33% compared to B2 at α = 10°. D2 shows a more significant change, with a maximum increase of 11.28% at α = 10°. The decrease in the number of corrugations has little influence on the ${\bar{C}}_{D}$ of the corrugated wing at most AOAs.

**Figure 20:**
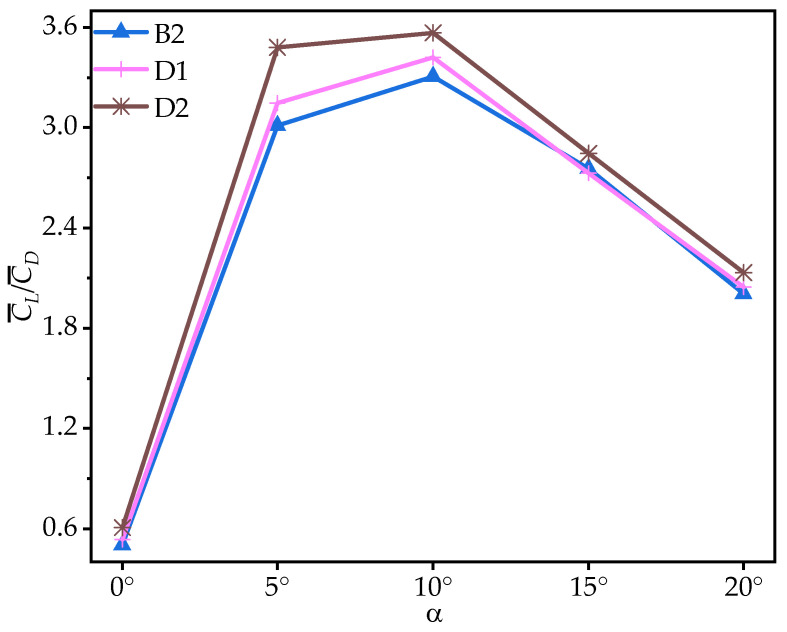
Variation in time-averaged lift-to-drag ratios (${\bar{C}}_{L}/{\bar{C}}_{D}$) of corrugated wings B2, D1, and D2 with the angle of attack (AOA).

From the foregoing analysis, it can be inferred that the variation in the number of trailing-edge corrugations significantly enhances ${\bar{C}}_{L}$, while the change in ${\bar{C}}_{D}$ is not notable at most AOAs. To further investigate the effect of the variation in the number of trailing-edge corrugations on aerodynamic performance, Figure 20 presents the time-averaged lift-to-drag ratios (${\bar{C}}_{L}/{\bar{C}}_{D}$) of corrugated wings B2, D1, and D2 at various AOAs. From Figure 20 and Table 6, it can be observed that as the number of corrugations decreases, the ${\bar{C}}_{L}/{\bar{C}}_{D}$ improves for both D1 and D2 across all AOAs. Compared to B2, the ${\bar{C}}_{L}/{\bar{C}}_{D}$ of D1 is slightly improved, ranging from 1.91% to 6.26%, while D2 shows a substantial increase, with a maximum improvement of 20.91%.

**Figure 21:**
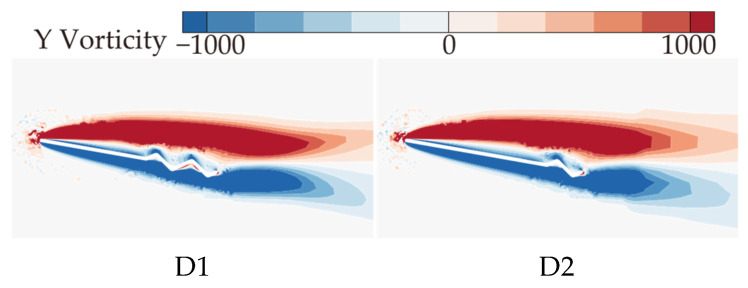
Time-averaged vorticity contours of corrugated wings D1 and D2 at 50% wingspan (α = 10°).

**Figure 22:**
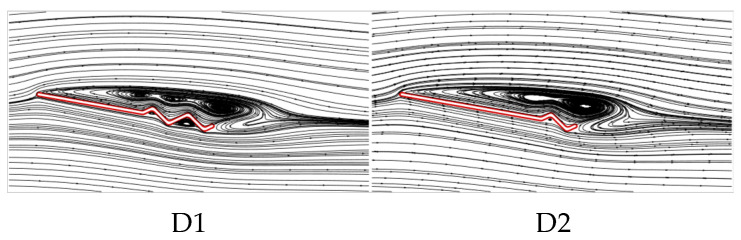
Time-averaged streamlines of corrugated wings D1 and D2 at 50% wingspan (α = 10°).

The above analysis of B2, D1, and D2 indicates that as the number of trailing-edge corrugations decreases, both the ${\bar{C}}_{L}$ and ${\bar{C}}_{L}/{\bar{C}}_{D}$ of the corrugated wings are further improved, with the best aerodynamic performance achieved when the number of corrugations is reduced to one (wing D2). This resembles the conclusion of Rohit [19], who observed that a reduction in the number of leading-edge corrugations can enhance airfoil aerodynamic performance. To analyze the effects of the number of corrugations on the flow field structure in more detail, time-averaged vorticity contour and streamlines at 50% wingspan for wings D1 and D2 are compared at α = 10°, where the aerodynamic force differences are most pronounced, as shown in Figure 21 and Figure 22. Combined with Figure 9 and Figure 10, it is evident that as the number of trailing-edge corrugations decreases, the degree of LEV shedding gradually slows, and the localized vortex between the leading edge and the first corrugation moves toward the trailing edge. In D2, the upper surface vortices merge completely, forming a larger and more coherent vortex at the trailing edge compared to those in C1 (Figure 15 and Figure 16), which is crucial for increasing the negative pressure on the upper surface. This phenomenon is consistent with Rohit’s [19] observation on reducing the number of leading-edge corrugations. He attributed it to the fact that a greater number of corrugation structures causes the leading-edge vortex to break up, disrupting its continuity. On the lower surface, as the number of corrugations decreases, both the number and strength of vortices within the grooves decrease, reducing the generation of negative pressure peaks on the lower surface.

**Figure 23:**
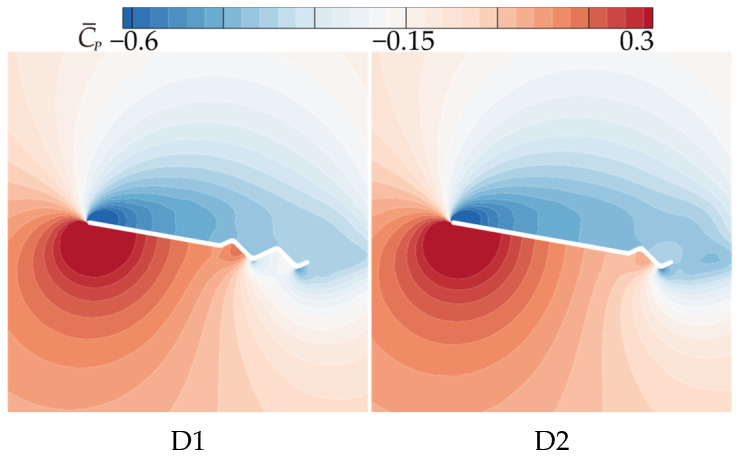
Time-averaged pressure coefficient contours of corrugated wings D1 and D2 at 50% wingspan (α = 10°).

**Figure 24:**
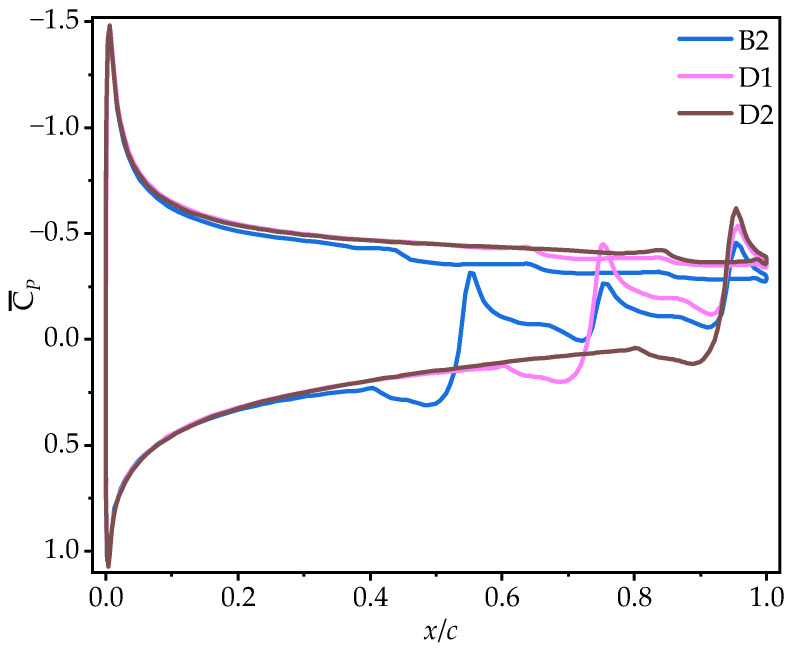
Time-averaged pressure coefficient curves of corrugated wings D1 and D2 at 50% wingspan (α = 10°).

The observations from the time-averaged pressure coefficient (${\bar{C}}_{P}$) contour plots in Figure 11 and Figure 23 support the above conclusions. As the number of trailing-edge corrugations decreases, the negative pressure region on the upper surface and the positive pressure region on the lower surface both increase gradually. The ${\bar{C}}_{P}$ curves in Figure 24 further demonstrate that as the number of corrugations decreases, the negative pressure in the middle-to-rear region of the upper surface increases. On the lower surface, the number of negative pressure peaks at the corrugations decreases, greatly enhancing the pressure difference between the upper and lower surfaces, which is the main reason for the improved aerodynamic performances of D1 and D2.

## 4. Conclusions

This study systematically investigates the aerodynamic effects of chordwise corrugation position, linear corrugation amplitude variation, and the number of trailing-edge corrugations on the gliding performance of three-dimensional wings at *Re* = 1350.

1. Trailing-edge corrugations (B2) significantly enhanced aerodynamic performance compared to leading-edge configurations (B1). The closer proximity of corrugations toward the trailing edge promoted stronger LEV adhesion to the upper surface, expanded the low-pressure region, and increased the lift coefficient by up to 9.70% relative to a flat plate (A) at α = 10°.
2. A linear increase in corrugation amplitude toward the trailing edge (C1) delayed LEV shedding and reduced vortex generation in lower-surface grooves. This configuration achieved a 28.99% increase in lift coefficient and a 31.96% improvement in lift-to-drag ratio compared to uniform-amplitude corrugations (B2).
3. Reducing the number of trailing-edge corrugations (e.g., D2 with one corrugation) consolidated localized vortices into larger, coherent LEVs, further increasing upper-surface suction. On the other hand, the number of negative pressure peaks at the corrugations decreases on the lower surface, thus enhancing the pressure difference between the upper and lower surfaces. D2 exhibited a 20.09% increase in lift coefficient and a 20.91% improvement in lift-to-drag ratio compared to B2.

These results demonstrate that the strategic chordwise placement and design of corrugated structures can optimize the aerodynamic performance in low-Reynolds-number gliding applications. Specifically, trailing-edge corrugations with a linear increase in amplitude and minimal corrugation numbers are highly effective for enhancing the lift-to-drag characteristics through an optimized flow field structure. This work provides actionable principles for the biomimetic design of MAV wings. However, this study focuses on gliding and a fixed Reynolds number, which may limit its direct applicability to dynamic flapping. Future studies could explore dynamic interactions between multiple corrugation parameters and their scalability across broader *Re* ranges.

## Figures and Tables

**Table 1:** Comparison between this study and prior research on corrugated wings.

	Researcher	Dimensionality	Corrugation	*Re*
Chordwise corrugation position	Xu [18]	2D	At leading, middle, and trailing edge	1500
	Meng [20]	3D	Close to the leading edge	1000
	Rohit [19]	2D	At leading and trailing edge	1000
	This study	3D	At leading and trailing edge	1350
Corrugation amplitude	Zhang [24]	2D	Global variation in all corrugation amplitudes	500~12,000
	Shabbir [25]	2D	Amplitude variation in the first two leading-edge corrugations	58,000, 125,000
	Wang [27]	3D	Global variation in corrugation amplitude	50,000~100,000
	This study	3D	Chordwise linear variation in corrugation amplitude	1350
Number of corrugations	Rohit [19]	2D	Variation in the number of leading-edge corrugations	1000
	This study	3D	Variation in the number of trailing-edge corrugations	1350

**Table 2:** Time-averaged lift coefficients (${\bar{C}}_{L}$), drag coefficients (${\bar{C}}_{D}$), and relative errors ($\Delta {\bar{C}}_{L}$, $\Delta {\bar{C}}_{D}$) under different time steps (α = 15°, *Re* = 1350).

Time Step (s)	${\bar{C}}_{L}$	($\Delta {\bar{C}}_{L}$)	${\bar{C}}_{D}$	($\Delta {\bar{C}}_{D}$)
2 × 10^−4^	0.756	1.05%	0.268	0.74%
1 × 10^−4^	0.764	0.13%	0.270	0%
5 × 10^−5^	0.763		0.270	

**Table 3:** Time-averaged lift coefficients (${\bar{C}}_{L}$), drag coefficients (${\bar{C}}_{D}$), and relative errors ($\Delta {\bar{C}}_{L}$, $\Delta {\bar{C}}_{D}$) of Mesh 1, Mesh 2, and Mesh 3 (α = 15°, *Re* = 1350).

	Mesh Numbers (Million)	${\bar{C}}_{L}$	($\Delta {\bar{C}}_{L}$)	${\bar{C}}_{D}$	($\Delta {\bar{C}}_{D}$)
Mesh 1	2.1	0.733	4.06%	0.261	3.33%
Mesh 2	5.0	0.764	2.55%	0.270	2.17%
Mesh 3	10.0	0.784		0.276	

**Table 4:** Rates of change in the time-averaged lift coefficients (${\bar{C}}_{L}$), drag coefficients (${\bar{C}}_{D}$), and lift-to-drag ratios (${\bar{C}}_{L}/{\bar{C}}_{D}$) of corrugated wings B1 and B2 relative to the flat plate wing A (the time-averaged lift coefficient of flat plate wing A at α = 0° is 0).

	Wing	0°	5°	10°	15°	20°
${\bar{C}}_{L}$	B1	↑	8.26% ↓	19.11% ↓	16.48% ↓	10.09% ↓
	B2	↑	8.49% ↑	9.70% ↑	2.79% ↑	12.84% ↓
${\bar{C}}_{D}$	B1	8.87% ↑	10.60% ↑	13.00% ↑	1.26% ↓	1.75% ↓
	B2	13.30% ↑	13.61% ↑	11.60% ↑	8.74% ↑	4.09% ↓
${\bar{C}}_{L}/{\bar{C}}_{D}$	B1	↑	17.06% ↓	28.41% ↓	15.41% ↓	8.49% ↓
	B2	↑	4.51% ↓	1.70% ↓	5.47% ↓	9.13% ↓

**Table 5:** Rates of change in the time-averaged lift coefficients (${\bar{C}}_{L}$), drag coefficients (${\bar{C}}_{D}$), and lift-to-drag ratios (${\bar{C}}_{L}/{\bar{C}}_{D}$) of corrugated wings C1, C2, and C3 relative to wing B2.

	Wing	0°	5°	10°	15°	20°
${\bar{C}}_{L}$	C1	28.99% ↑	11.76% ↑	10.85% ↑	7.53% ↑	5.06% ↑
	C2	52.72% ↓	10.24% ↓	6.50% ↓	10.55% ↓	7.63% ↑
	C3	13.42% ↓	0.33% ↑	0.69% ↓	1.81% ↓	7.01% ↑
${\bar{C}}_{D}$	C1	2.25% ↓	0.26% ↓	6.77% ↑	4.22% ↑	1.45% ↑
	C2	6.85% ↓	7.76% ↓	7.93% ↓	10.39% ↓	2.46% ↑
	C3	5.87% ↓	5.21% ↓	2.87% ↓	3.68% ↓	2.48% ↑
${\bar{C}}_{L}/{\bar{C}}_{D}$	C1	31.96% ↑	12.05% ↑	3.83% ↑	3.17% ↑	3.56% ↑
	C2	49.77% ↓	2.69% ↓	1.55% ↑	0.18% ↓	5.05% ↑
	C3	8.03% ↓	5.84% ↑	2.25% ↑	1.94% ↑	4.41% ↑

**Table 6:** Rates of change in the time-averaged lift coefficients (${\bar{C}}_{L}$), drag coefficients (${\bar{C}}_{D}$), and lift-to-drag ratios (${\bar{C}}_{L}/{\bar{C}}_{D}$) of corrugated wings D1 and D2 relative to wing B2.

	Wing	0°	5°	10°	15°	20°
${\bar{C}}_{L}$	D1	5.84% ↑	3.76% ↑	10.00% ↑	3.32% ↑	1.34% ↑
	D2	19.84% ↑	17.39% ↑	20.09% ↑	15.11% ↑	9.51% ↑
${\bar{C}}_{D}$	D1	0.49% ↓	0.62% ↓	6.33% ↑	1.16% ↑	0.56% ↓
	D2	0.88% ↓	1.60% ↑	11.28% ↑	8.10% ↑	3.07% ↑
${\bar{C}}_{L}/{\bar{C}}_{D}$	D1	6.26% ↑	4.41% ↑	3.45% ↑	2.14% ↑	1.91% ↑
	D2	20.91% ↑	15.54% ↑	7.92% ↑	6.49% ↑	6.25% ↑

## Data Availability

The original contributions presented in this study are included in the article; further inquiries can be directed to the corresponding author.
